# Cardio-hepatic syndrome in patients undergoing transcatheter aortic valve replacement

**DOI:** 10.1007/s00392-023-02245-w

**Published:** 2023-06-19

**Authors:** Lukas Stolz, Michael Kirchner, Julius Steffen, Philipp M. Doldi, Daniel Braun, Ludwig T. Weckbach, Thomas J. Stocker, Kornelia Löw, Julius Fischer, Magda Haum, Hans D. Theiss, Konstantinos Rizas, Martin Orban, Sven Peterß, Michael Näbauer, Steffen Massberg, Jörg Hausleiter, Simon Deseive

**Affiliations:** 1 Medizinische Klinik und Poliklinik I, Klinikum der Universität München, Marchioninistr. 15, 81377 Munich, Germany; 2 German Center for Cardiovascular Research (DZHK), Partner Site Munich Heart Alliance, Munich, Germany; 3 Herzchirurgische Klinik und Poliklinik, Klinikum der Universität München, Munich, Germany

**Keywords:** Cardiohepatic syndrome, Liver function, Aortic stenosis, Congestion, Heart failure, TAVR

## Abstract

**Background:**

Cardiohepatic syndrome (CHS) has been identified as an important but underrecognized survival predictor in multiple cardiovascular disease entities. The objectives of this study were to evaluate the prevalence and prognostic value of CHS in patients undergoing TAVR for severe aortic stenosis (AS).

**Methods:**

The study included patients with available laboratory parameters of hepatic function who underwent TAVR from July 2013 until December 2019 at our center. CHS was defined as an elevation of at least two of three laboratory cholestasis parameters above the upper limit of normal (bilirubin, alkaline phosphatase, and gamma glutamyl transferase). Study endpoints were three-year survival, technical and device failure (VARC 3), as well as New York Heart Association (NYHA) functional class at follow-up.

**Results:**

Among a total of 953 analyzed patients (47.6% females, median age 80.0 [76.0–85.0] years) CHS was present in 212 patients (22.4%). In patients with vs. without CHS, rates of technical (6.1% vs. 8.4%, p = 0.29) and device failure (18.9% vs. 17.3%, p = 0.59) were comparable. NYHA functional class at baseline and follow-up was more severe in patients with CHS. Nevertheless, heart failure symptoms improved from baseline to follow-up irrespective of hepatic function. Three-year survival rates were significantly lower in patients with CHS (49.4 vs. 65.4%, p < 0.001). The predictive value of CHS persisted after adjustment in a multivariable analysis (hazard ratio 1.58, p < 0.01).

**Conclusion:**

In patients undergoing TAVR, CHS is prevalent in 22% of patients and is associated with increased postinterventional mortality. Thus, CHS should be included in the decision-making process within the TAVR heart team.

**Graphical abstract:**

Cardiohepatic syndrome (CHS) as defined by an elevation of at least two of three laboratory cholestasis parameters above the upper limit of normal was prevalent in 22% of patients undergoing TAVR for severe AS. The presence of CHS was associated with more severe heart failure symptoms and worse three-year survival. 
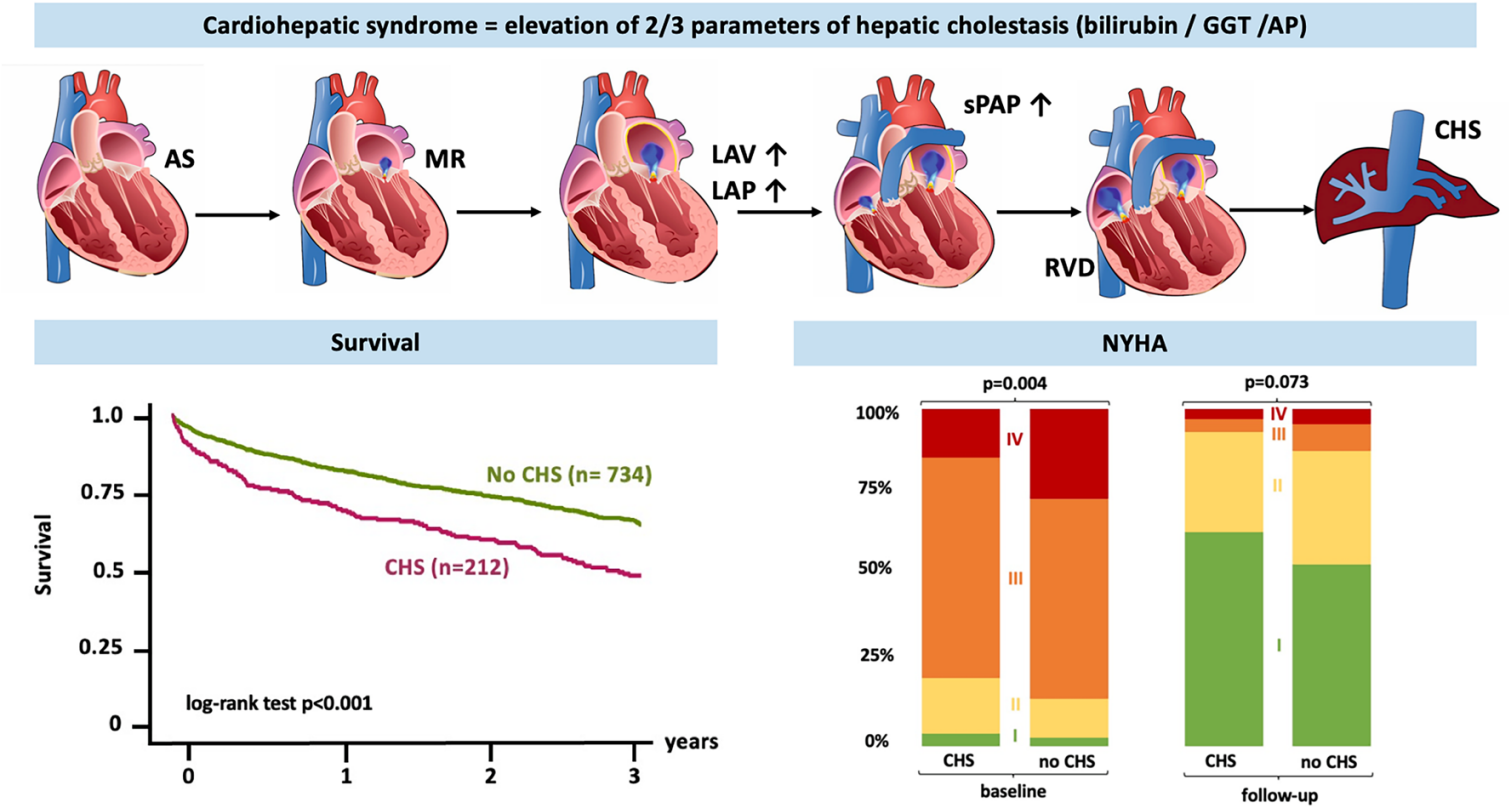

**Supplementary Information:**

The online version contains supplementary material available at 10.1007/s00392-023-02245-w.

## Introduction

Within the past two decades, transcatheter aortic valve replacement (TAVR) has become an indispensable pillar in the treatment of severe aortic stenosis (AS). Since multiple clinical trials and registries have proven the safety and effectiveness of treating severe AS by TAVR [1, 2], research is now heading towards gaining a deeper pathophysiological understanding of this heterogeneous disease entity [3] in order to further optimize patient selection and treatment.

After being underrecognized for years, the crucial prognostic importance of right ventricular (RV) function and tricuspid regurgitation (TR) has been demonstrated for multiple cardiologic disease entities, such as heart failure with preserved ejection fraction [4], mitral regurgitation (MR) [5, 6], and AS [7]. In the context of TAVR, right ventricular dysfunction (RVD) is commonly a consequence of long-standing pressure overload of the left ventricle and thus increased filling pressure of the left atrium which might lead to mitral regurgitation and pulmonary hypertension. As soon as the RV is no longer capable of adapting to an excessively increasing afterload, RV dilation occurs which has been shown to be associated with impaired survival prognosis after TAVR [8]. Ultimately, the pathophysiological cascade described above can lead to chronic systemic venous congestion with corresponding end-organ damage [9, 10] (Graphic Abstract). Apart from the rather well characterized cardio-renal syndrome [11], the relationship of hepatic function and heart failure in TAVR patients is less well studied.

Recently, we identified the presence of the cardiohepatic syndrome (CHS) to be associated with impaired survival prognosis after transcatheter tricuspid and mitral valve edge-to-edge repair [12]. In concordance with preexisting literature, the latter was as defined by an elevation of two out of three laboratory parameters of hepatic cholestasis [13, 14].

The aim of this study was to investigate whether the application of CHS could contribute to improved risk stratification and mortality prediction in TAVR patients. We therefore evaluated the impact of CHS on three-year survival and symptomatic outcome in a large retrospective cohort of patients who underwent TAVR for severe symptomatic aortic stenosis.

## Methods

### Study cohort, treatment and follow-up

This study included patients who underwent transfemoral TAVR for severe symptomatic aortic stenosis from July 2013 until December 2019 at Munich University Hospital, Ludwig-Maximilians-Universität (LMU Munich, Germany). CHS was defined as an elevation of two out of three parameters of hepatic cholestasis (bilirubin, gamma glutamyl transferase [GGT] and alkaline phosphatase [AP]) [12, 13]. Patients with missing information on more than one laboratory parameters were excluded (Supplementary Table 1). TAVR was performed following an interdisciplinary heart team consensus considering age, comorbidities, and life expectancy.

TAVR procedures were performed using a transfemoral access under local anesthesia as described in previous literature [15]. Unfractionated heparin (50–70 IE/kg body weight) or bivalirudin were used for periprocedural anticoagulation. The decision to perform pre- and post-dilation was left to the interventionalist’s discretion. Suture mediated or and/or plug-based closure devices were used for femoral access hemostasis.

Follow-up included regular visits in the outpatient department of our clinic and phone calls with the patients, their local practitioners or the next of kin. Mortality follow-up was completed by using the national death registry. The present study was approved by the ethics committee of the LMU University Hospital and adheres to the principles outlined in the declaration of Helsinki.

### Study endpoints

The primary endpoint of the present study was three-year survival. Secondary endpoints comprised symptomatic improvement as represented by New York Heart Association function class (NYHA), and the Valve Academic Research Consortium (VARC-3) composite endpoints device failure at 30 days and technical failure.

### Study variables

Clinical study variable included sex, age, body mass index (BMI), Society of Thoracic Surgeons (STS) Score, previous myocardial infarction (MI), previous coronary artery bypass grafting (CABG), previous percutaneous coronary intervention (PCI), atrial fibrillation (Afib), chronic obstructive pulmonary disease (COPD), arterial hypertension (AHT), diabetes mellitus (DM), need of chronic hemodialysis, NYHA functional class, heart failure medication and laboratory parameters of hepatic function.

Echocardiography was performed by experienced cardiologists in line with recent guidelines [16, 17]. The recorded parameters included left ventricular ejection fraction (LVEF), left ventricular end-diastolic diameter (LVEDD), left atrial volume (LAV), stroke volume index (SVi), mean systolic aortic valve pressure gradient (AV dPmean), aortic valve opening area (AVA), tricuspid annular plane systolic excursion (TAPSE), maximum systolic tricuspid pressure gradient (TrMaxPg), RV basal diameter (RVbase).

**Fig. 1:**
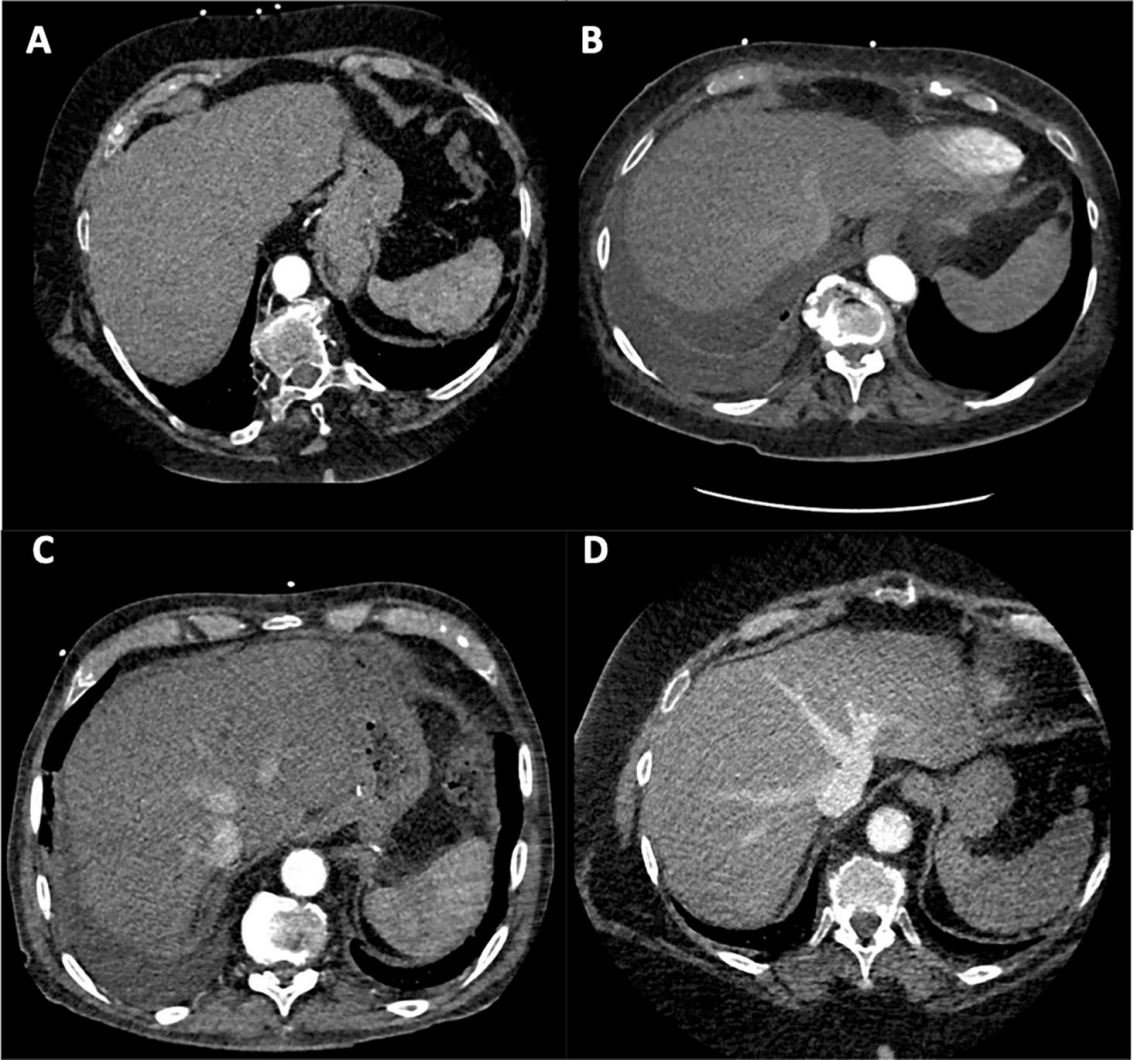
Visual quantification of contrast agent backflow into the hepatic veins. **A**–**D** Represent no (0), mild (1), moderate (2) and severe (3) contrast agent backflow into the hepatic veins during the arterial imaging phase

To further quantify right-ventricular dysfunction as a cause of CHS, backflow of contrast agent into the hepatic veins during pre-interventional computed tomography (CT) was used. Backflow was semiquantitatively graded (0: none, 1: mild, 2: moderate; 3: severe; Fig. 1).

### Hepatic function and cardiohepatic syndrome

Baseline laboratory evaluation of hepatic function included total bilirubin, aspartate aminotransferase (AST), alanine aminotransferase (ALT), alkaline phosphatase (AP), gamma glutamyltransferase (GGT), albumin and cholinesterase (CHE). Bilirubin was considered abnormal if exceeding 1.2 mg/dL, irrespective of sex. For AST, ALT, GGT, and AP, we used sex-specific laboratory cut-off values: for AST and ALT, > 34 U/L (female) and > 49 U/L (male); for GGT, > 39 U/L (female) and 59 U/L (male); and for AP, 105 U/L (female) and 130 U/L (male). In line with previous literature, CHS was defined as elevation of at least two of three laboratory cholestasis parameters above the upper limit of normal (bilirubin, AP, and GGT). As stated above, patients with missing values of more than one parameter of hepatic cholestasis (bilirubin, AP, GGT) were excluded from the study. In the case of one missing value, the corresponding parameter was assumed not to be elevated in favor of a conservative assessment of CHS.

### Statistics

Normality of data distribution was tested graphically and using the Kolmogorov–Smirnov and Shapiro–Wilk tests. Continuous study variables were depicted as mean ± standard deviation (sd) or median with interquartile range, accordingly. Nominal and ordinal data were presented as counts and proportions. Differences of two independent samples were tested using Mann–Whitney U test or Pearson chi-square test, as appropriate. The Wilcoxon test was applied when comparing two dependent samples. Survival differences were depicted by Kaplan–Meier curves and analyzed using the log-rank test. A Cox regression model was built to identify predictors for three-year all-cause mortality. Parameters with p < 0.05 in the univariate analysis were included into multivariate backwards elimination model. Results are shown as hazard ratio (HR) with 95% confidence interval (CI) and the respective p-value. A two-sided p-value of p < 0.05 was defined as threshold of statistical significance. Statistical analyses were performed using SPSS version 25 (IBM) and R version 4.0.4 (R Foundation for Statistical Computing).

## Results

### Study characteristics and outcome

The present study included 953 patients (47.6% females, median age 80.0 [76.0–85.0] years, Table 1A). Against the background of an overall preserved left ventricular function (LVEF of 55.0 [44.8–57.0] %), patients presented with a median AVA, SVi and dPmean of 0.74 [0.60–0.88] cm^2^, 33.9 [27.4–41.2] ml and 36.0 [26.0–45.0] mmHg, respectively. 167 patients (26.9%) suffered from RVD defined as TAPSE < 17 mm. Concomitant MR ≥ 2 + and TR ≥ 3 + were observed in 27.4% and 5.3% of patients, respectively. As indicated by a median STS score of 4.0 [2.4–7.1] %, the majority of patients presented with advanced or prohibitive surgical risk. Contrast agent reflux was absent, mild, moderate, and severe in 58.3%, 24.3%, 12.0% and 5.4%, respectively. Table 1A–C summarize clinical and echocardiographic baseline characteristics of the study cohort.

**Table 1:** Baseline characteristics stratified by the presence of CHS

	All patients (n = 953)	CHS (n = 212)	No CHS (n = 741)	p-value
**1A. Clinical characteristics**				
Female sex	454 (47.6)	104 (49.1)	391 (52.8)	0.639
Age, years	80 [76.0–85.0]	80 [75.0–84.0]	81 [76.0–85.0]	0.011
BMI, kg/cm^2^	25.6 [23.1–29.1]	25.5 [22.5–28.5]	25.6 [23.3–29.3]	0.131
STS-score, %	4.0 [2.4–7.1]	5.7 [3.0–9.8]	3.8 [2.3–6.7]	< 0.001
CAD	610 (64.0)	123 (58.0)	487 (65.7)	0.039
Previous MI	167 (18.0)	35 (17.0)	132 (18.2)	0.670
Previous CABG	96 (10.2)	15 (7.1)	81 (11.1)	0.103
Previous PCI	339 (36.1)	70 (33.8)	269 (36.7)	0.446
Afib/flutter	298 (31.3)	95 (44.8)	203 (27.4)	< 0.001
COPD	173 (18.2)	42 (19.8)	131 (17.7)	0.478
DM	295 (31.0)	69 (32.5)	226 (30.5)	0.570
AHT	865 (90.8)	187 (88.2)	678 (91.5)	0.145
Previous SAVR	82 (8.6)	26 (12.3)	56 (7.6)	0.031
NYHA class				
I	19 (3.4)	3 (2.7)	16 (3.6)	0.004
II	87 (15.5)	13 (11.5)	74 (6.6)	
III	360 (64.3)	67 (59.3)	293 (65.5)	
IV	94 (16.8)	30 (26.5)	64 (14.3)	
**1B. Medication**				
BB	268 (48.0)	82 (57.3)	186 (42.4)	0.002
OAC	256 (32.2)	80 (50.3)	176 (29.4)	< 0.001
Diuretics	302 (52.1)	88 (62.0)	214 (48.9)	0.007
**1C. Laboratory values**				
Hemoglobin, g/dl	12.1 [10.5–13.3]	11.9 [10.1–13.3]	12.1 [10.6–13.4]	0.170
Creatinine, mg/dl	1.2 [1.0–1.6]	1.4 [1.0–1.9]	1.1 [1.0–1.5]	< 0.001
Bilirubin, mg/dl	0.7 [0.5–1.0]	1.3 [0.7–1.8]	0.7 [0.5–0.9]	< 0.001
AST, U/l	27.0 [21.0–36.0]	34.5 [27.8–48.3]	25 [20.0–32.0]	< 0.001
ALT, U/l	20.0 [14.0–29.0]	27.0 [19.0–46.0]	18.0 [13.0–27.0]	< 0.001
GGT, U/l	43.0 [24.0–93.0]	129.0 [77.5–216.0]	34.0 [21.0–58.0]	< 0.001
AP, U/l	83.0 [65.0–111.0]	135.0 [113.0–177.0]	75.0 [61.0–91.0]	< 0.001
**1D. Echocardiography**				
LVEF, %	55.0 [43.0–57.0]	47.5 [38.0–55.0]	55.0 [45.0–58.0]	< 0.001
LVEDD, mm	48.0 [42.0–54.0]	49.0 [44.0–55.0]	47.0 [42.0–52.0]	0.040
AVA, cm^2^	0.74 [0.60–0.88]	0.74 [0.59–0.90]	0.74 [0.60–0.88]	0.890
dPmean, mmHg	36.0 [26.0–45.0]	29.0 [21.0–41.0]	37.0 [28.0–46.0]	< 0.001
SVi, ml/cm^2^	33.9 [27.4–41.1]	30.7 [22.8–37.9]	34.8 [28.3–41.8]	< 0.001
TrMaxPG, mmHg	36.0 [27.0–45.0]	39.0 [29.0–47.0]	35.0 [27.0–44.0]	0.097
TAPSE, mm	20.0 [16.0–24.0]	18.0 [15.0–23.0]	20.0 [17.0–24.0]	< 0.001
TR severity				
0+	93 (12.3)	13 (8.0)	80 (13.5)	< 0.001
1+	498 (66.0)	88 (54.7)	410 (69.0)	
2+	124 (16.4)	41 (25.5)	83 (14.0)	
3+	28 (3.7)	10 (6.2)	18 (3.0)	
4+	12 (1.6)	9 (5.6)	3 (0.5)	
MR severity				
0+	72 (8.6)	10 (5.6)	62 (9.5)	< 0.001
1+	535 (64.0)	99 (55.0)	436 (66.5)	
2+	162 (19.4)	48 (26.7)	114 (17.4)	
3+	55 (6.6)	17 (9.4)	38 (5.8)	
4+	12 (1.4)	6 (3.3)	6 (0.9)	
Type of AS				
HG	329 (44.7)	52 (34.7)	277 (47.3)	0.185
cLFLG	177 (24.0)	53 (35.3)	124 (21.1)	
pLFLG	120 (16.3)	28 (18.7)	92 (15.7)	
NFLG	110 (14.9)	17 (11.3)	93 (15.7)	
**1E. Computed tomography**				
Hepatic vein reflux				
0	501 (58.3)	73 (39.5)	428 (63.5)	< 0.001
1	209 (24.3)	52 (28.1)	157 (23.3)	
2	103 (12.0)	34 (18.4)	69 (10.2)	
3	46 (5.4)	26 (14.1)	20 (3.0)	

*AHT* arterial hypertension, *ALT* alanine aminotransferase, *AP* alkaline phosphatase, *AS* aortic stenosis, *AST* aspartate aminotransferase, *AVA* aortic valve opening area, *BB* beta blocker, *BMI* body mass index, *CABG* coronary artery bypass grafting, *CAD* coronary artery disease, *cLFLG* classical low flow low gradient, *COPD* chronic obstructive pulmonary disease, *DM* diabetes mellitus, *dPmean* mean pressure gradient, *GGT* gamma glutamyltransferase, *HG* high gradient, *LVEDD* left ventricular end diastolic diameter, *LVEF* left ventricular ejection fraction, *MI* myocardial infarction, *MR* mitral regurgitation, *NFLG* normal flow low gradient, *NYHA* New York Heart Association functional class, *OAC* oral anticoagulation therapy, *PCI* percutaneous coronary intervention, *pLFLG* paradox low flow low gradient, *SAVR* surgical aortic valve replacement, *STS* society of thoracic surgeons score, *SVi* stroke volume index, *TAPSE* tricuspid annular plane systolic excursion, *TR* tricuspid regurgitation, *TrMaxPG* maximum transtricuspid pressure gradient

Completeness of survival follow-up at three years was 88%. Technical and device failure occurred in 7.9% and 17.6% of patients, respectively. At latest available follow-up, the percentage of patients presenting with NYHA class III or IV significantly decreased compared to baseline (83.2% vs. 7.8%, p < 0.001). Details regarding procedural and clinical outcomes are displayed in Table 2.

**Table 2:** Procedural and clinical outcomes

	All patients (n = 953)	CHS (n = 212)	No CHS (n = 741)	p-value
Technical failure	75 (7.9)	13 (6.1)	62 (8.4)	0.287
Device failure	168 (17.6)	40 (18.9)	128 (17.3)	0.591
NYHA class at follow-up				
I	345 (61.6)	61 (54.0)	284 (63.5)	0.037
II	171 (30.5)	38 (33.6)	133 (29.8)	
III	26 (4.6)	9 (8.0)	17 (3.8)	
IV	18 (3.2)	5 (4.4)	13 (2.9)	

*CHS* cardiohepatic syndrome, *NYHA class* New York Heart Association functional class

### Hepatic function and cardiohepatic syndrome

**Fig. 2:**
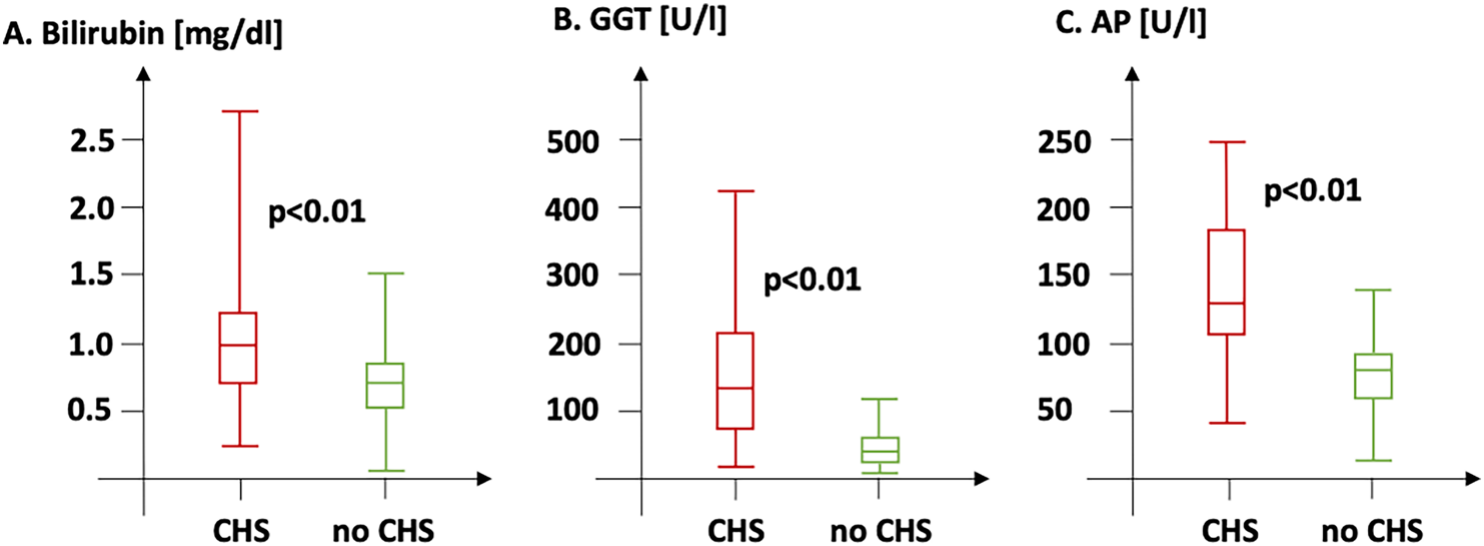
Laboratory parameters of hepatic cholestasis in patients with vs. without CHS. Serums levels of bilirubin (**A**), GGT (**B**) and AP (**C**) were significantly higher in the presence of CHS. *AP* alkaline phosphatase, *CHS* cardiohepatic syndrome, *GGT* gamma glutamyltransferase

Laboratory assessment of hepatic function (Table 1C) indicated overall normal levels of transaminases (GOT 27.0 [21.0–33.0] U/l; GPT 20.0 [14.0–26.0] U/l) and parameters of hepatic cholestasis (bilirubin 0.70 [0.50–1.0] mg/dl; GGT 43 [24.0–92.0]; AP 83.0 [65.0–111.0], Fig. 2). CHS as defined by an elevation of at least two of three laboratory cholestasis parameters above the upper limit of normal was present in 212 patients (22.4%).

As shown in Table 1D, CHS was associated with larger left ventricular dimensions (49.0 vs. 47.0 mm, p = 0.040), more severe concomitant MR (≥ 2+: 11.8% vs. 3.5% p < 0.001), numerically higher transtricuspid pressure gradients (39 vs. 35 mmHg, p = 0.097) and worse TAPSE (18 vs. 20 mm, p < 0.001). While AVA was comparable in patients with vs. without CHS (0.74 vs. 0.74 cm^2^, p = 0.890), impaired hepatic function was associated with a lower dPmean (29.0 vs. 37.0 mmHg, p < 0.001). The prevalence of CHS varied depending on the type of AS and was most frequently observed in patients with classical low-flow low-gradient AS (cLFLG, 29.9%) followed by paradox low-flow low-gradient AS (pLFLG, 23.3%) classical high-grade AS (HG AS, 15.8%) and normal flow low-gradient AS (NFLG, 15.5%). Beyond that, severe contrast agent reflux was observed significantly more often in patients with CHS compared to those without (14.1 vs. 3.0%, p < 0.001, Table 1E).

### Prognostic implications of the cardio hepatic syndrome

**Fig. 3:**
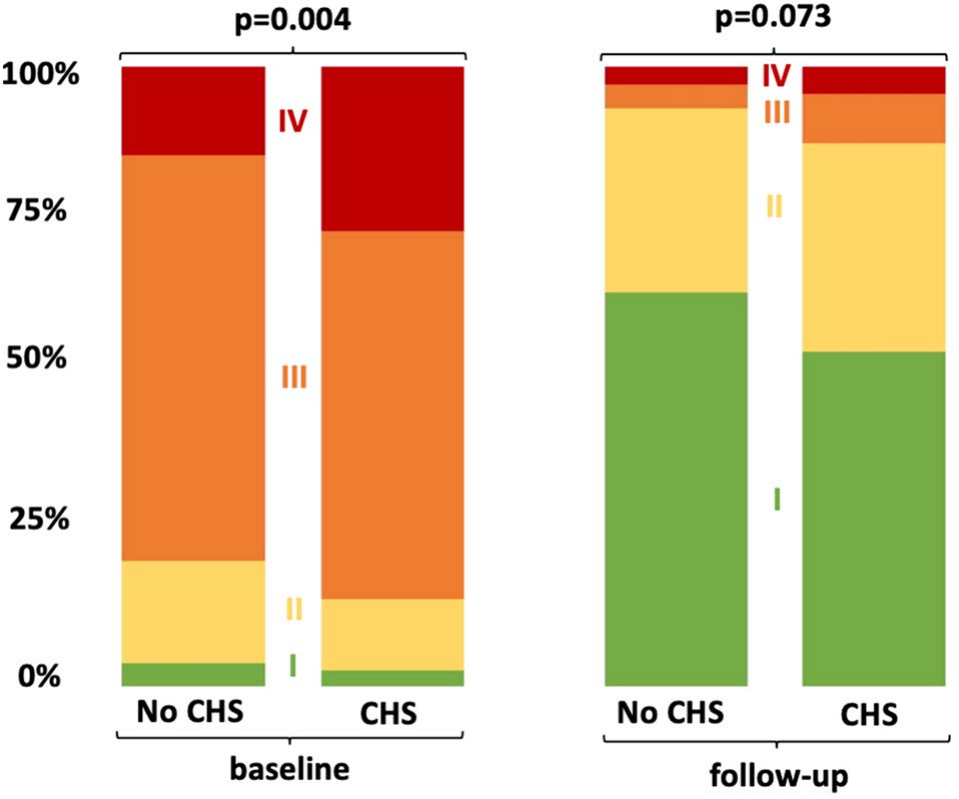
Development of NYHA functional class after TAVR stratified by liver function. CHS was associated with more severe heart failure symptoms at baseline and follow-up. Nevertheless, NYHA functional class improved irrespective of the presence of CHS at baseline. *CHS* cardiohepatic syndrome, *NYHA* New York Heart Association functional class

Rates of technical (6.1 vs. 8.4%, p = 0.287) and device failure (18.9 vs. 17.3%, p = 0.591) did not differ in patients with and without CHS. Even though patients with CHS suffered from more severe heart failure symptoms at baseline and follow-up (Fig. 3), NYHA class significantly improved irrespective of hepatic function.

**Fig. 4:**
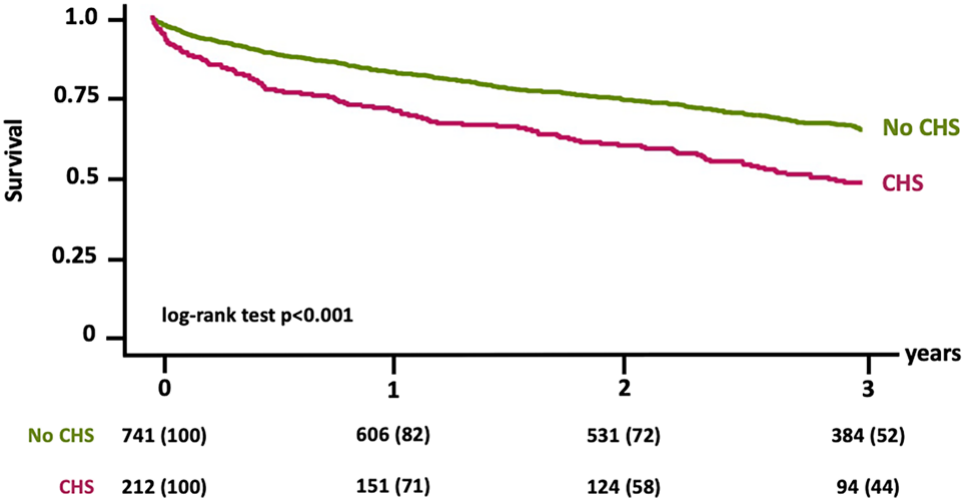
Impact of CHS on survival outcome after TAVR. Patients with CHS presented with significantly worse one-, two- and three-year survival rates compared to those with normal hepatic function. *CHS* cardiohepatic syndrome

The presence of CHS was associated with significantly reduced one-, two-and three-year survival rates after TAVR (1y: 70.8 vs. 82.3%, 2y: 60.2 vs. 74.4%, 3y: 44.4 vs. 65.4%, for patients with vs. without CHS all p < 0.001, Fig. 4). Besides hemoglobin levels (HR 0.842; CI 0.779–0.910, p < 0.001), COPD (HR 1.620; CI 1.134–2.315; p = 0.008), male sex (HR 1.708; CI 1.226–2.318; p = 0.002) and LVEF < 35% (HR 1.651; CI 1.039–2.623; p = 0.034) CHS was a significant multivariate predictor for three-year all-cause mortality within the overall study cohort (HR 1.580; CI 1.122–2.225; p = 0.009) (Table 3, Supplementary Table 2). Of note transaminases did not provide prognostic value in terms of three-year all-cause mortality. Beyond that, Supplementary Table 3 shows differences in laboratory liver parameters stratified by three-year survivors vs. non-survivors in our study cohort.

**Table 3:** Multivariate Cox regression model (summary)

	Univariate			Multivariate		
	Hazard ratio	Confidence interval	p-value	Hazard ratio	Confidence interval	p-value
Sex, male	1.470	1.187–1.821	< 0.001	1.708	1.226–2.318	0.002
COPD	1.361	1.060–1.748	0.016	1.620	1.134–2.315	0.008
CHS	1.707	1.359–2.144	< 0.001	1.580	1.122–2.225	0.009
Hemoglobin	0.834	0.789–0.881	< 0.001	0.842	0.779–0.910	< 0.001
LVEF < 35%	2.031	1.519-2.714	< 0.001	1.651	1.039–2.623	0.034

*CHS* cardiohepatic syndrome, *COPD* chronic obstructive pulmonary disease, *LVEF* left ventricular ejection fraction

## Discussion

### Overview

In the present study we applied the definition of CHS as elevation of two out of three laboratory parameters of hepatic cholestasis [12, 13] to a large cohort of patients who underwent TAVR for AS. The main findings of our study were (Graphic Abstract):

1. *CHS has an overall prevalence of 22.4% in patients undergoing TAVR*
2. *CHS is associated with worse three-year survival after TAVR*
3. *CHS is associated with more severe heart failure symptoms at baseline and follow-up*
4. *TAVR leads to symptomatic improvement irrespective of baseline CHS*

### Pathophysiology

As described previously, long standing aortic stenosis leads to a cascade of unfavorable pathologic processes [18, 19]. In an initial phase of the disease, concentric left ventricular hypertrophy enables an adequate response to the AS-induced increase in afterload. When being left untreated, LV function deteriorates, and remodeling of the ventricle occurs [18]. Over the course of the disease, patients might suffer from increasing LA pressures, atrial fibrillation and diastolic dysfunction. Some patients might additionally suffer from subsequent mitral regurgitation. The above-mentioned processes may finally lead to the development of postcapillary pulmonary hypertension. Consequently, the RV dilates, and RV function decreases while secondary tricuspid regurgitation can be observed. The latter leads to venous backflow into the right atrium and both, the superior and inferior caval veins enlarge. Chronic systemic venous congestion finally leads to end-organ damage, among them the development of cardiohepatic syndrome [10, 13].

The results underline the concept of a left-sided obstruction resulting in pulmonary congestion and, finally, RV dysfunction. In comparison to patients with normal hepatic function, CHS was associated with more severe LV dilation, reduction in LVEF, higher prevalence of concomitant MR ≥ 2+ and atrial fibrillation. Additionally, patients with CHS presented with more severely impaired TAPSE and higher rates of concomitant TR. It should be noted that the concept described here is a theoretical construct and probably does not apply to every patient. Not all patients with RVD and hepatic congestion present with MR and postcapillary pulmonary hypertension. In some cases, RVD may also be intrinsic or be the result of pulmonary disease (e.g. precapillary pulmonary hypertension or chronic obstructive pulmonary disease). To further evaluated this, CT contrast agent backflow was semi-quantitatively evaluated. The degree of contrast agent backflow was found to be more pronounced in patients with CHS.

### CHS in the setting of TAVR and its prognostic implications

With a prevalence of 22.4% in the overall study population, CHS was expectably less frequent compared to patients with TR undergoing transcatheter edge-to-edge repair (T-TEER, 45.2%), which is not surprising considering the congestive stress due to significant venous backflow in the setting of severe TR [12]. CHS was most often observed in patients with classical low-flow low-gradient aortic stenosis (29.9%) compared to paradox low-flow low-gradient AS (23.3%) or high gradient AS (15.8%). This might primarily be due to the fact that patients with classic low-flow low-gradient AS suffer from more severely reduced LV systolic function and have higher rates of concomitant TI and RV dysfunction.

Taken together, the diagnosis of CHS could be an indicator of a very advanced overall process of underlying valvular heart disease. The fact that CHS maintained its predictive value against important echocardiographic and clinical parameters in a multivariate Cox analysis suggests that its diagnosis involves more information than simply that of advanced RV dysfunction, which has also been shown in patients undergoing T-TEER for symptomatic TR [12]. Of note, it should be emphasized that with a 3-year survival follow-up rate of approximately 90% the results of the present study are based on a solid data fundament.

Concordantly, CHS was also associated with more pronounced heart failure symptoms at baseline and follow-up as represented by more severe NYHA functional class compared to patients with normal hepatic function. Of note, significant symptomatic improvement was achieved irrespective of the presence of CHS at baseline. That fits well with the finding that rates of device and technical failure did not differ between patients with and without CHS.

This is of particular importance given that neither current guidelines, nor established risk scores incorporate liver function in their decision making and risk assessment [17]. While the impact of liver function is not included in the EuroScore II or German AV risk calculator, the STS risk score only mentions the presence of liver disease without further definition.

The current study is subject to some limitations, mainly due to its retrospective nature. The necessity to exclude patients without available parameters of hepatic function might have biased the results to some extent. Nevertheless, this is the largest TAVR cohort with detailed information on hepatic cholestasis in a significant number of patients. Of note, no information on pulmonary hypertension was available for the current analysis.

CHS is associated with a more progressive disease state in patients undergoing TAVR for severe AS and is associated with significantly impaired three-year survival. Nevertheless, TAVR provides symptomatic benefit irrespective of hepatic function and needs to be considered within the heart team.

### Supplementary Information

Below is the link to the electronic supplementary material.

Supplementary file 1 (DOCX 26 KB)

## Data Availability

The datasets generated and analyzed during the current study are available from the corresponding author on reasonable request.
