# Supplementary material for: Cardio-hepatic syndrome in patients undergoing transcatheter aortic valve replacement
Source: Clin Res Cardiol. 2023 Jun 19;112(10):1427–35. doi: 10.1007/s00392-023-02245-w (PMC10562337; doi:10.1007/s00392-023-02245-w)
Supplement: Supplementary file 1 — (DOCX 26 KB) [file 392_2023_2245_MOESM1_ESM.docx]

**SUPPLEMENTARY INFORMATION**

| **Supplementary Table 1. Characteristics of in- and excluded patients** | | | |
| --- | --- | --- | --- |
|  | **included**  (n=953) | **excluded**  (n=1976) | **p-value*** |
| **S1A. Clinical characteristics** | | | |
| Sex, female | 454 (47.6) | 989 (50.1) | 0.241 |
| Age, years | 80 [76.0-85.0] | 82 [78.0-86.0] | <0.001 |
| BMI, kg/cm^2^ | 25.6 [23.1-29.1] | 25.9 [23.4-28.9] | 0.255 |
| STS-Score, % | 4.0 [2.4-7.1] | 3.7 [2.1-5.6] | <0.001 |
| CAD | 610 (64.0) | 1223 (61.9) | 0.268 |
| Previous MI | 167 (18.0) | 290 (15.2) | 0.057 |
| Previous CABG | 96 (10.2) | 182 (9.4) | 0.493 |
| Previous PCI | 339 (36.1) | 687 (35.4) | 0.747 |
| Afib/flutter | 298 (31.3) | 550 (27.8) | 0.055 |
| COPD | 173 (18.2) | 294 (14.9) | 0.023 |
| DM | 295 (31.0) | 610 (30.9) | 0.963 |
| AHT | 865 (90.8) | 1799 (91.0) | 0.807 |
| Previous SAVR | 82 (8.6) | 81 (4.1) | <0.001 |
| NYHA class  I  II  III  IV | 19 (3.4)  87 (15.5)  360 (64.3)  94 (16.8) | 44 (3.3)  188 (14.0)  1041 (77.5)  71 (5.3) | <0.001 |
| **S1B. Medication** | | | |
| BB | 268 (48.0) | 570 (42.7) | 0.652 |
| OAC | 256 (32.2) | 494 (30.7) | 0.115 |
| Diuretics | 302 (52.1) | 581 (48.2) | 0.127 |
| **S1C. Laboratory values** | | | |
| Hemoglobin, g/dl | 12.1 [10.5-13.3] | 12.6 [11.4-13.7] | <0.001 |
| Creatinine, mg/dl | 1.2 [1.0-1.6] | 1.1 [0.9-1.4] | <0.001 |
| Bilirubin, mg/dl | 0.7 [0.5-1.0] | 0.7 [0.5-0.8] | 0.214 |
| AST, U/l | 27.0 [21.0-36.0] | 27.0 [22.0-33.0] | 0.215 |
| ALT, U/l | 20.0 [14.0-29.0] | 19.0 [14.0-26.0] | 0.039 |
| GGT, U/l | 43.0 [24.0-93.0] | 38.0 [22.0-71.0] | <0.001 |
| AP, U/l | 83.0 [65.0-111.0] | 73.5 [66.0-73.5] | 0.539 |
| **S1D. Echocardiography** | | | |
| LVEF, % | 55.0 [43.0-57.0] | 55.0 [48.0-58.0] | <0.001 |
| LVEDD, mm | 48.0 [42.0-54.0] | 46.0 [42.0-52.0] | 0.070 |
| AVA, cm^2^ | 0.74 [0.60-0.88] | 0.74 [0.60-0.89] | 0.250 |
| dPmean, mmHg | 36.0 [26.0-45.0] | 37.0 [28.0-46.0] | 0.031 |
| SVi, ml/cm^2^ | 33.9 [27.4-41.1] | 35.6 [29.5-42.6] | <0.001 |
| TrMaxPG, mmHg | 36.0 [27.0-45.0] | 34.0 [27.0-45.0] | 0.178 |
| TAPSE, mm | 20.0 [16.0-24.0] | 21.0 [17.0-24.0] | <0.001 |
| TR severity  0+  1+  2+  3+  4+ | 93 (12.3)  498 (66.0)  124 (16.4)  28 (3.7)  12 (1.6) | 290 (17.5)  1133 (68.4)  181 (10.9)  41 (2.5)  12 (0.7) | <0.001 |
| MR severity  0+  1+  2+  3+  4+ | 72 (8.6)  535 (64.0)  162 (19.4)  55 (6.6)  12 (1.4) | 233 (13.0)  1237 (68.8)  259 (14.4)  58 (3.2)  11 (0.6) | <0.001 |
| Type of AS  HG  cLFLG  pLFLG  NFLG | 329 (44.7)  177 (24.0)  120 (16.3)  110 (14.9) | 760 (46.9)  307 (19.0)  265 (16.4)  288 (17.8) | 0.827 |
| **S1E. Computed tomography** | | | |
| Hepatic vein reflux  0  1  2  3 | 501 (58.3)  209 (24.3)  103 (12.0)  46 (5.4) | 1197 (67.9)  357 (20.2)  159 (9.0)  51 (2.9) | <0.001 |
| **S1F. Outcomes** | | | |
| Technical failure | 65 (6.8) | 88 (4.5) | 0.007 |
| Device failure | 158 (16.6) | 239 (12.1) | <0.001 |
| NYHA class follow-up  I  II  III  IV | 345 (61.6)  171 (30.5)  26 (4.6)  18 (3.2) | 798 (59.4)  464 (34.5)  53 (3.9)  29 (2.2) | 0.589 |
| HT = arterial hypertension; ALT = alanine aminotransferase; AP = alkaline phosphatase; AS = aortic stenosis; AST = aspartate aminotransferase; AVA = aortic valve opening area; BB= beta blocker; BMI = body mass index; CABG = coronary artery bypass grafting; CAD = coronary artery disease; cLFLG = classical low flow low gradient; COPD = chronic obstructive pulmonary disease; DM = diabetes mellitus; dPmean = mean pressure gradient; GGT = gamma glutamyltransferase; HG = high gradient; LVEDD = left ventricular end diastolic diameter; LVEF = left ventricular ejection fraction; MI = myocardial infarction; MR = mitral regurgitation; NFLG = normal flow low gradient; NYHA = New York Heart association functional class; OAC = oral anticoagulation therapy; PCI = percutaneous coronary intervention; pLFLG = paradox low flow low gradient; SAVR = surgical aortic valve replacement; STS = society of thoracic surgeons score; SVi = stroke volume index; TAPSE = tricuspid annular plane systolic excursion; TR = tricuspid regurgitation; TrMaxPG = maximum transtricuspid pressure gradient | | | |

| **Supplementary Table 2. Multivariate Cox regression model (details)** | | | | | | |
| --- | --- | --- | --- | --- | --- | --- |
|  | **univariate** | | | **multivariate** | | |
|  | **hazard ratio** | **confidence interval** | **p-value** | **hazard ratio** | **confidence interval** | **p-value** |
| Sex, female | **1.470** | **1.187-1.821** | **<0.001** | **1.708** | **1.226-2.318** | **0.002** |
| Age, years | **1.023** | **1.009-1.037** | **0.001** |  |  |  |
| BMI, kg/cm^2^ | 0.989 | 0.969-1.010 | 0.304 |  |  |  |
| CAD | **1.301** | **1.039-1.629** | **0.022** |  |  |  |
| Previous MI | **1.421** | **1.102-1.832** | **0.007** | * | * | * |
| Previous CABG | 1.235 | 0.888-1.717 | 0.209 |  |  |  |
| Previous PCI | 1.105 | 0.890-1.372 | 0.367 |  |  |  |
| Afib/flutter | **1.819** | **1.471-2.248** | **<0.001** |  |  |  |
| COPD | **1.361** | **1.060-1.748** | **0.016** | **1.620** | **1.134-2.315** | **0.008** |
| DM | 1.181 | 0.947-1.472 | 0.139 |  |  |  |
| AHT | 0.987 | 0.690-1.413 | 0.945 |  |  |  |
| Previous SAVR | 0.944 | 0.642-1.389 | 0.770 |  |  |  |
| Beta blocker | 0.865 | 0.665-1.124 | 0.278 |  |  |  |
| OAC | **1.066** | **1.002-1.135** | **0.044** |  |  |  |
| Diuretic medication | 1.184 | 0.910-1.540 | 0.209 |  |  |  |
| CHS | **1.707** | **1.359-2.144** | **<0.001** | **1.580** | **1.122-2.225** | **0.009** |
| NYHA IV | 1.169 | 0.859-2.373 | 0.169 |  |  |  |
| Hemoglobin | **0.834** | **0.789-0.881** | **<0.001** | **0.842** | **0.779-0.910** | **<0.001** |
| eGFR < 60 ml/min | **1.843** | **1.379-2.463** | **<0.001** |  |  |  |
| LVEF < 35% | **0.972** | **0.963-0.982** | **<0.001** | **1.651** | **1.039-2.623** | **0.034** |
| LVEDD, mm | 1.033 | 0.969-1.101 | 0.316 |  |  |  |
| AVA, cm^2^ | 0.904 | 0.535-1.527 | 0.707 |  |  |  |
| dPmean, mmHg | **0.981** | **0.973-0.989** | **<0.001** |  |  |  |
| SVi, ml/cm^2^ | **0.974** | **0.962-0.986** | **<0.001** | ***** | ***** | ***** |
| TAPSE < 17 mm | **0.953** | **0.930-0.978** | **<0.001** |  |  |  |
| TR ≥ 3+ | **2.373** | **1.584-3.556** | **<0.001** |  |  |  |
| MR ≥ 3+ | **1.832** | **1.305-2.571** | **<0.001** |  |  |  |
| AHT = arterial hypertension; AVA = aortic valve opening area; BMI = body mass index; CABG = coronary artery bypass grafting; CAD = coronary artery disease; CHS = Cardiohepatic syndrome; COPD = chronic obstructive pulmonary disease; DM = diabetes mellitus; dPmean = mean pressure gradient; eGFR = estimated glomerular filtration rate; LVEDD = left ventricular end diastolic diameter; LVEF = left ventricular ejection fraction; MI = myocardial infarction; MR = mitral regurgitation; NYHA = New York Heart Association; PCI = percutaneous coronary intervention; SVi = stroke volume index; SAVR = surgical aortic valve replacement; STS = society of thoracic surgeons score; TAPSE = tricuspid annular plane systolic excursion; TR = tricuspid regurgitation; *excluded due to duplication of information | | | | | | |

| **Supplementary Table 3. Laboratory liver parameters stratified by three-year survival** | | | |
| --- | --- | --- | --- |
|  | Three-year  survivors | Three-year  non-survivors | p-value |
| Bilirubin, mg/dl | 0.7 [0.5-1.0] | 0.8 [0.5-1.1] | 0.014 |
| AST, U/l | 26.0 [21.0-34.0] | 28.0 [21.0-39.0] | 0.032 |
| ALT, U/l | 19.0 [14.0-28.0] | 20.0 [14.0-32.0] | 0.183 |
| GGT, U/l | 37.0 [22.0-73.0] | 60.0 [31.0-143.0] | <0.001 |
| AP, U/l | 77.0 [63.0-103.0] | 94.0 [70.0-129.0] | <0.001 |
| ALT = alanine aminotransferase; AST = aspartate aminotransferase; AP = alkaline phosphatase; GGT = gamma glutamyltransferase | | | |
